# Supplementary material for: Category learning can alter perception and its neural correlates
Source: PLoS One. 2019 Dec 6;14(12):e0226000. doi: 10.1371/journal.pone.0226000 (PMC6897555; doi:10.1371/journal.pone.0226000)
Supplement: S1 File — Supplementary analysis by block of Experiments 1 and 2. (DOCX) [file pone.0226000.s001.docx]

**Appendix – Analysis by block**

In our before-after ERP analysis, we compared the across-subject means for the trials before and after reaching the learning criterion. This method has the following shortcomings, noted in the Discussion: (1) learning may be gradual rather than all-or-none and (2) the number of trials before vs. after learning is unequal in almost all cases. Subjects reached criterion at different points throughout the category learning phase, ranging from the 80^th^ to the 340^th^ of the 400 training trials. This imbalance can increase variance as well as add noise to the averaged ERP for Learners. (Non-Learners’ data were always evenly split between their first and last 200 trials.) For a more continuous picture of the ERP correlates of category learning we did a second analysis comparing each of the four successive 100-trial four blocks. This could be done in the same way for successful Learners, borderlines and Non-Learners.

**Experiment 1**

This second analysis showed a progressive decrease in the occipital N1 negativity from the 1st block to the 4^th^ block in our Learners; this effect was absent in the Non-Learners (Figure A1). We also found a progressive increase in the LPC for Learners, but not for Non-Learners (Figure A2). A between-subjects repeated measures ANOVA to test the effect of learning on these changes (N1 and LPC amplitude) showed a significant interaction between learning group (Learner vs. Non-Learner) and the LPC change across the blocks but failed to show this same interaction for the N1 change throughout the blocks. However, within-subjects repeated-measures ANOVAs showed that both the N1 and the LPC changed significantly for the Learners, while these significant effects were absent in the Non-Learners and Borderlines (Table A1).


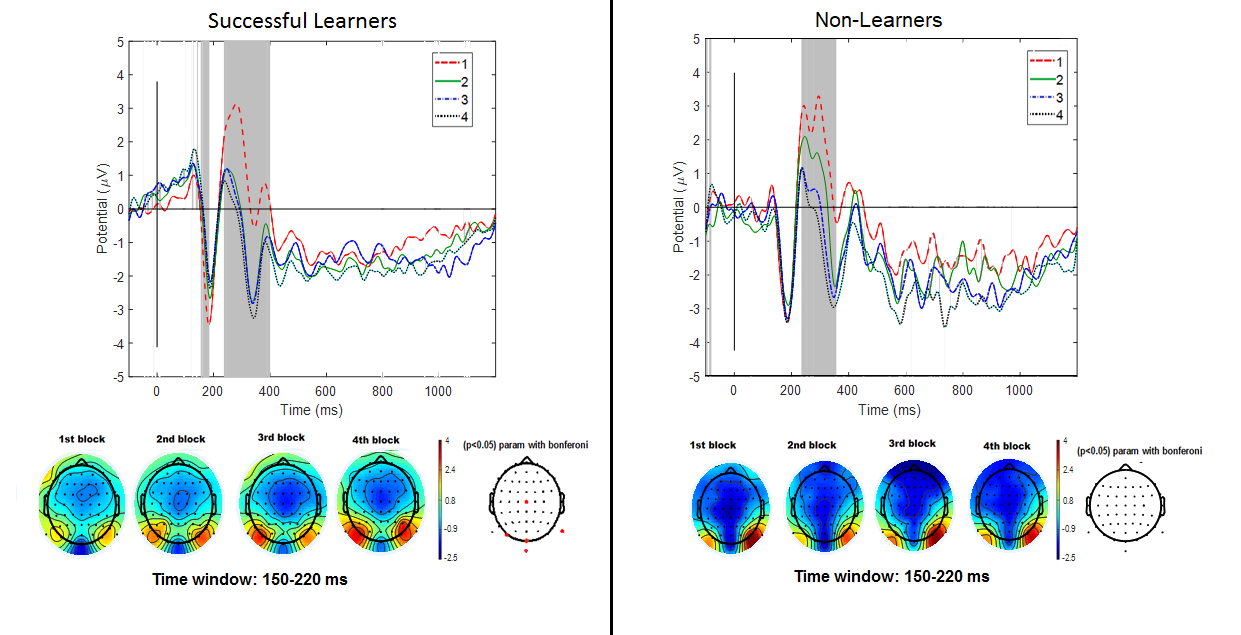


**S1 Fig****. ERP waveforms and scalpmaps for the four successive 100-trial blocks in Experiment 1 (occipital electrodes O1, O2, Oz and Iz):** In Learners (left) there is a significant progressive decrease in N1 negativity, absent in Non-Learners (right).


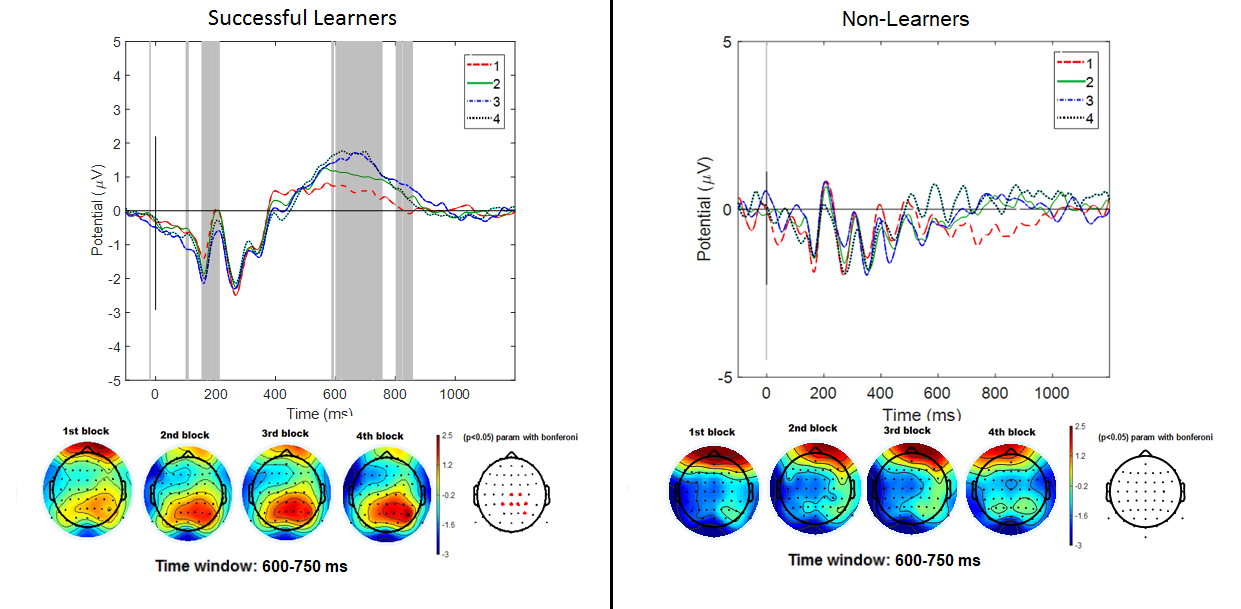
 **S2 Fig. ERP waveforms and scalp maps for the four successive 100-trial blocks in Experiment 1 (parietal electrodes and LPC windows):** In Learners (left) there is a significant progressive increase in LPC positivity, absent in Non-Learners (right).

*Table A1. Repeated-Measures ANOVAs for N1 and LPC amplitudes throughout the four blocks.*

|  |  | **N1 amplitude** | | | | **LPC amplitude** | | | |  |  |  |  |  |
| --- | --- | --- | --- | --- | --- | --- | --- | --- | --- | --- | --- | --- | --- | --- |
| Group | *Wilks Lambda* | *F* | *Df* | *P* | *Partial η2* | *Wilks Lambda* | *F* | *Df* | *p* | *Partial η2* |  |  |  |  |
| Successful  Learners | 0.308 | 2.823 | 3,20 | **0.056** | 0.308 | 0.676 | 2.881 | 3,20 | **0.065** | 0.324 |  |  |  |  |
| Non-Learners | 0.531 | 1.766 | 3,7 | 0.253 | 0.469 | 0.401 | 2.489 | 3,7 | 0.175 | 0.599 |  |  |  |  |
| Borderline | 0.030 | 10.82 | 3,2 | 0.219 | 0.970 | 0.195 | 2.759 | 3,2 | 0.277 | 0.805 |  |  |  |  |
| Between-Ss ANOVA | F(3,29)=1.811, p=0.188, ***partial η2***=0.054 | | | | | F(3,29)=5.473, p=0.026, ***partial η2***=0.159 | | | | |  |  |  | F(1,28)=4.258, p=0.042, ***partial η2***=0.139 |

Paired sample t-tests were applied to the changes between pairs of blocks (1^st^ vs. 2^nd^, 2^nd^ vs 3^rd^ vs. 4^th^) to determine which block transition showed the biggest effect. Visual inspection of the ERP waveforms in Figure A1 suggests that the biggest change in the Learners’ N1 component happened between the first and the second block. However, this difference did not prove significant; nor did the differences between the 2nd and 3rd block, or the 3rd and the 4th. The only significant difference was between the first and the last block, by which time all of the Learners had reached criterion (Table A2). For the LPC component, both the changes between the first and the second block and the changes between the first and the last block reached significance (Table A3).

*Table A2. Paired samples t-tests for N1 changes between progressive blocks.*

|  | **Successful Learners** | | | | | **Non-Learners** | | | | | | | |
| --- | --- | --- | --- | --- | --- | --- | --- | --- | --- | --- | --- | --- | --- |
| Block | *Mean* | | *t* | *Df* | *p* | | *d* |  | *Mean* | *T* | *df* | *P* | *d* |
| First - last | -1.688 | -0.491 | | 21 | **<0.001** | | **0.861** |  | 0.2540 | -1.330 | 8 | 0.220 | 0.448 |
| 1^st^ - 2^nd^ | -0.923 | -1.769 | | 21 | 0.091 | | 0.376 |  | -0.458 | -0.254 | 8 | 0.085 | 0.081 |
| 2^nd^ - 3^rd^ | -0.352 | -0.626 | | 21 | 0.538 | | 0.163 |  | -0.395 | -1.021 | 8 | 0.337 | 0.345 |
| 3^rd^ - 4^th^ | -0.215 | -0.491 | | 21 | 0.628 | | 0.173 |  | -0.162 | -0.531 | 8 | 0.610 | 0.184 |

*Table A3. Paired samples t-tests for LPC changes between progressive blocks.*

|  | **Successful Learners** | | | | | **Non-Learners** | | | | | | | |
| --- | --- | --- | --- | --- | --- | --- | --- | --- | --- | --- | --- | --- | --- |
| Block | *Mean* | | *t* | *Df* | *p* | | *d* |  | *Mean* | *T* | *df* | *P* | *d* |
| First - last | -1.422 | -2.786 | | 20 | **0.011** | | **0.633** |  | -1.103 | -1.554 | 8 | 0.159 | 0.335 |
| 1^st^ - 2^nd^ | -0.875 | -3.421 | | 20 | **0.003** | | **0.859** |  | -0.085 | -0.189 | 8 | 0.855 | 0.063 |
| 2^nd^ - 3^rd^ | -0.313 | -1.074 | | 20 | 0.296 | | 0.234 |  | -0.774 | -2.010 | 8 | 0.079 | 0.770 |
| 3^rd^ - 4^th^ | -0.194 | -0.825 | | 20 | 0.418 | | 0.192 |  | -0243 | -1.554 | 8 | 0.159 | 0.278 |

**Experiment 2**

We did the same analyses for Experiment 2, with much the same results (Figure A3 and A4): For N1, a repeated-measures ANOVA confirmed that the decrease in the amplitude across blocks was significant for the Learners, but not for the Non-Learners (Table A5); the same pattern was evident for the LPC amplitude.


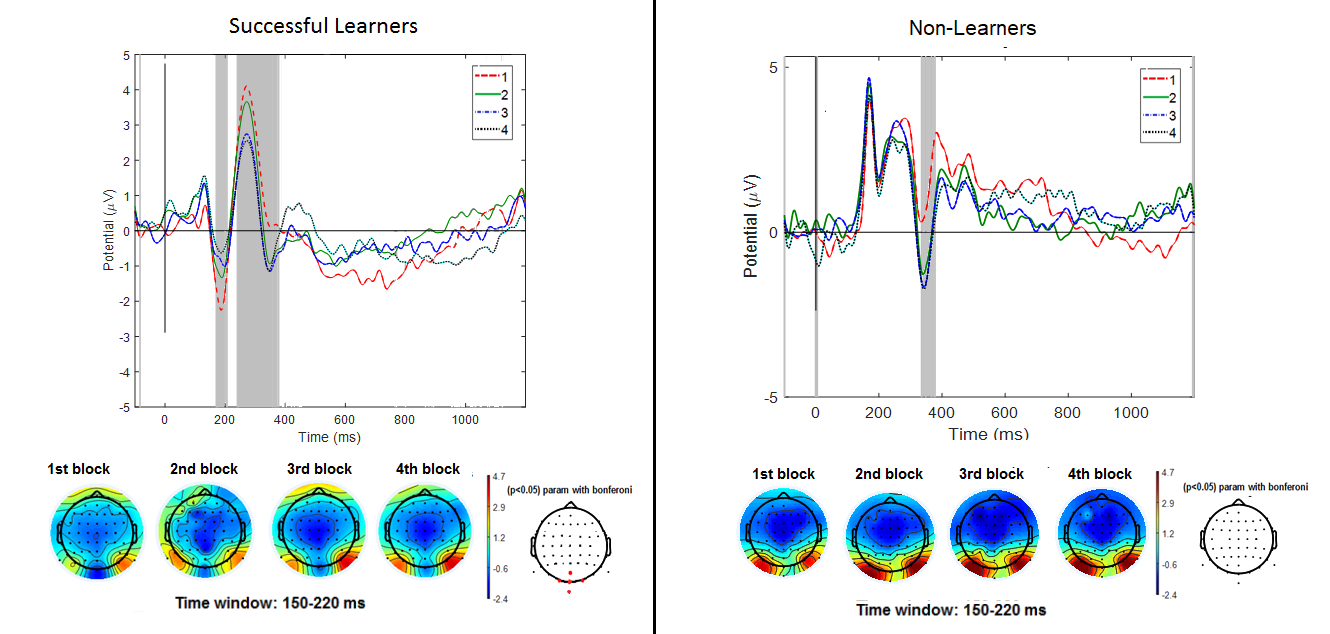


**Figure A3:** **Above**: ERP waveforms for the four successive 100-trial blocks in Experiment 2, in a cluster of occipital electrodes (O1, O2, Oz and Iz). On the left (Learners) there is a progressive, significant decrease in the N1 component for the Learners; on the right (Non-Learners) this effect is absent. **Below**: Scalp maps for each block, in the time window between 150-220 ms for Learners (left) and Non-Learners (right). Electrodes with significant changes in this time-window are highlighted in red.


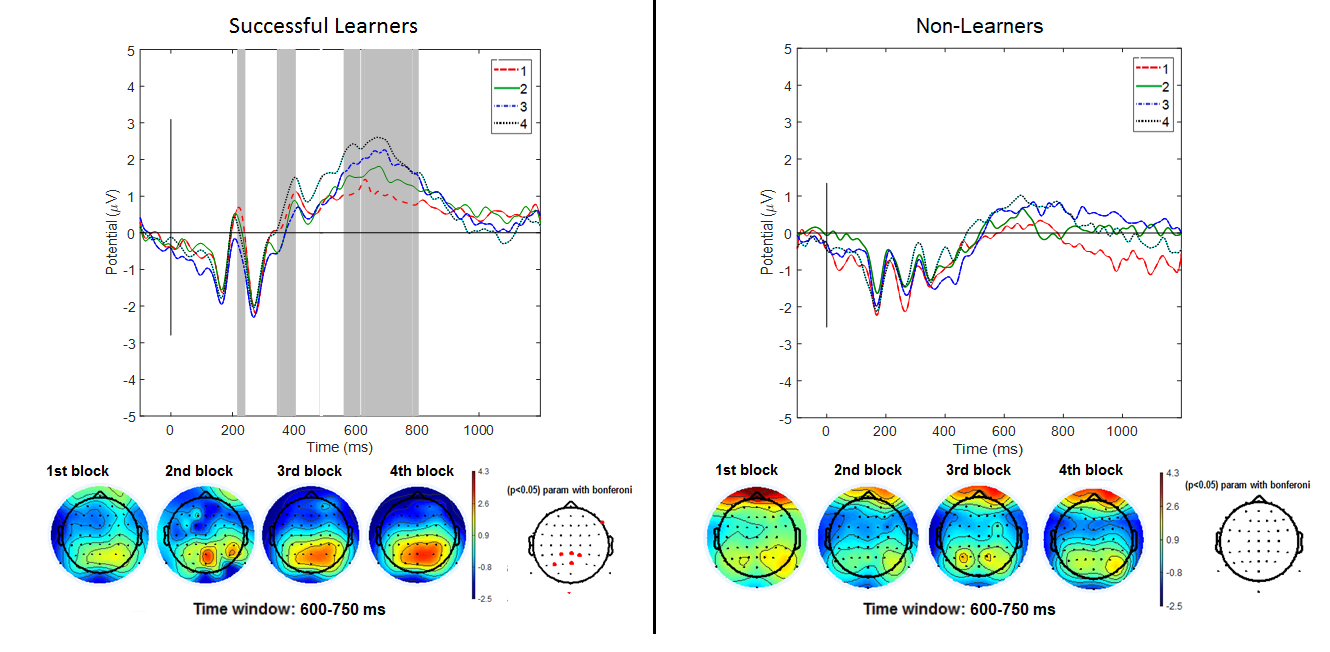


**Figure A4**: ERP waveforms for each 100-trial block in Experiment 2 ( parietal electrodes:Pz, P1,P2, CPz, CP1, CP2). In Learners (left) there is a significant progressive increase in LPC positivity, absent in the Non-Learners (right). **Below**: Scalp maps for each block, in the time window between 600-750 ms for Learners (left) and Non-Learners (right). Electrodes with significant changes in this time-window are highlighted in red.

*Table A4. Repeated-Measures ANOVAs for N1 peaks and amplitudes throughout the four blocks.*

|  |  | **N1 amplitude** | | | | **LPC amplitude** | | | |  |
| --- | --- | --- | --- | --- | --- | --- | --- | --- | --- | --- |
| Group | *Wilks Lambda* | *F* | *Df* | *p* | *Partial η2* | *Wilks Lambda* | *F* | *df* | *p* | *Partial η2* |
| Successful  Learners | 0.597 | 3.376 | 3,15 | **0.046** | 0.403 | 0.328 | 10.25 | 3,15 | **0.001** | 0.672 |
| Non-Learners | 0.942 | 0.226 | 3,11 | 0.877 | 0.058 | 0.873 | 0.533 | 3,11 | 0.669 | 0.127 |
| Between-Ss ANOVA | F(1,30) = 3.359, p = 0.468, *partial η2= 0.018* | | | | | F(1,30)=3.359, p=0.077, *partial η2*=0.101 | | | | |

T-tests for N1 peak pairs in successive blocks showed a significant difference between the first and the last block (Table A6), while T-tests for LPC peaks and amplitudes in successive blocks showed a significant difference between the first and the last block as well as between the first and the second block (Table A8).

*Table A5. Paired samples t-tests for N1 changes between progressive blocks.*

|  | **Successful Learners** | | | | | **Non-Learners** | | | | | | | |
| --- | --- | --- | --- | --- | --- | --- | --- | --- | --- | --- | --- | --- | --- |
| Block | *Mean* | | *t* | *Df* | *p* | | *D* |  | *Mean* | *T* | *df* | *P* | *d* |
| First - last | -1.015 | -2.117 | | 17 | **0.049** | | **0.521** |  | 0.2540 | -0.529 | 13 | 0.606 | 0.141 |
| 1^st^ - 2^nd^ | -0.952 | -1.932 | | 17 | 0.070 | | 0.462 |  | -0.458 | -0.920 | 13 | 0.374 | 0.245 |
| 2^nd^ - 3^rd^ | 0.058 | -0.130 | | 17 | 0.898 | | 0.031 |  | -0.395 | -0.218 | 13 | 0.831 | 0.059 |
| 3^rd^ - 4^th^ | -0.121 | -0.322 | | 17 | 0.751 | | 0.087 |  | -0.162 | 0.857 | 13 | 0.407 | 0.230 |

*Table A6. Paired samples t-tests for LPC changes between progressive blocks.*

|  | **Successful Learners** | | | | | **Non-Learners** | | | | | | | |
| --- | --- | --- | --- | --- | --- | --- | --- | --- | --- | --- | --- | --- | --- |
| Block | *Mean* | | *t* | *Df* | *p* | | *d* |  | *Mean* | *t* | *df* | *P* | *d* |
| First - last | -1.168 | -4.827 | | 17 | **0.001** | | **1.150** |  | -0.733 | -1.163 | 13 | 0.266 | 0.311 |
| 1^st^ - 2^nd^ | -0.7113 | -2.941 | | 17 | **0.009** | | **0.703** |  | -0.466 | -0.872 | 13 | 0.399 | 0.243 |
| 2^nd^ - 3^rd^ | -0.256 | -0.747 | | 17 | 0.465 | | 0.181 |  | 0.126 | -0.298 | 13 | 0.770 | 0.095 |
| 3^rd^ - 4^th^ | -0.2019 | -0.733 | | 17 | 0.474 | | 0.215 |  | -1.235 | -1.260 | 13 | 0.239 | 0.342 |
