# Supplementary figures and images for: Category learning can alter perception and its neural correlates
Source: PLoS One. 2019 Dec 6;14(12):e0226000. doi: 10.1371/journal.pone.0226000 (PMC6897555; doi:10.1371/journal.pone.0226000)

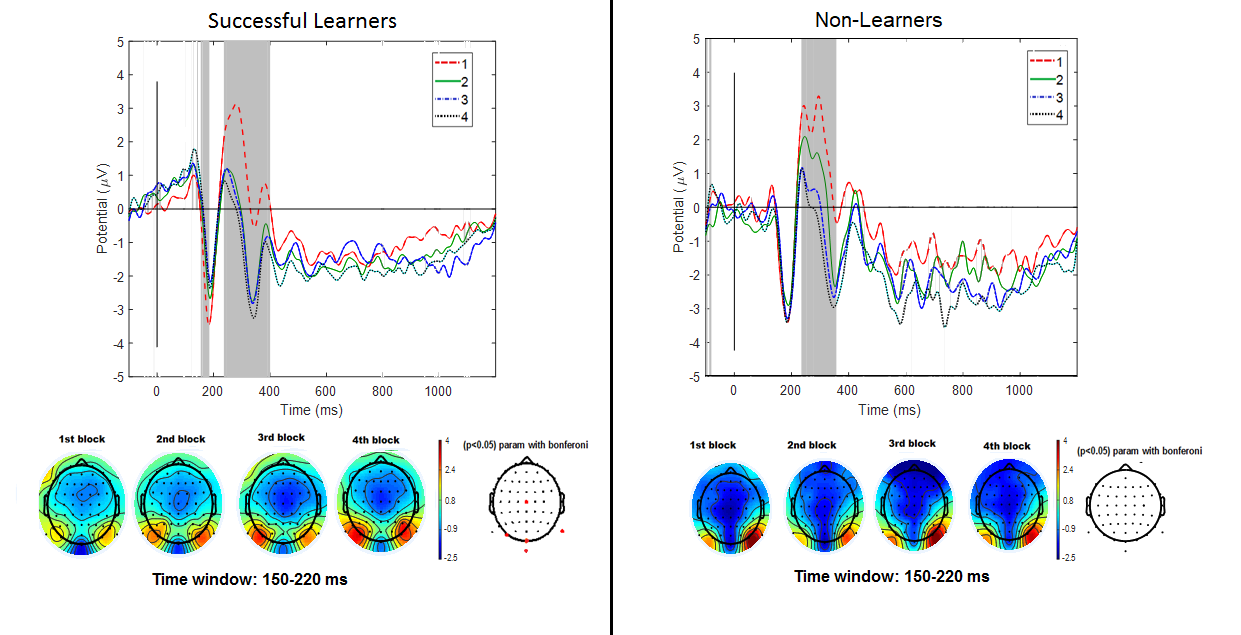

Supplement: S1 Fig — (TIF) [file pone.0226000.s002.tif]

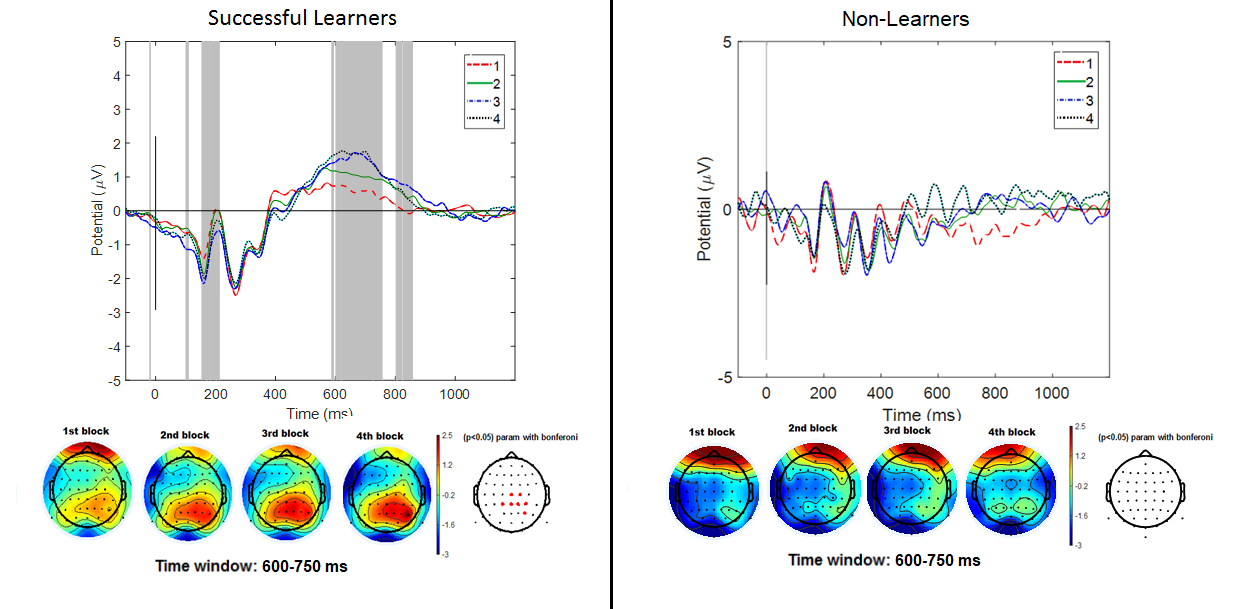

Supplement: S2 Fig — (TIF) [file pone.0226000.s003.tif]

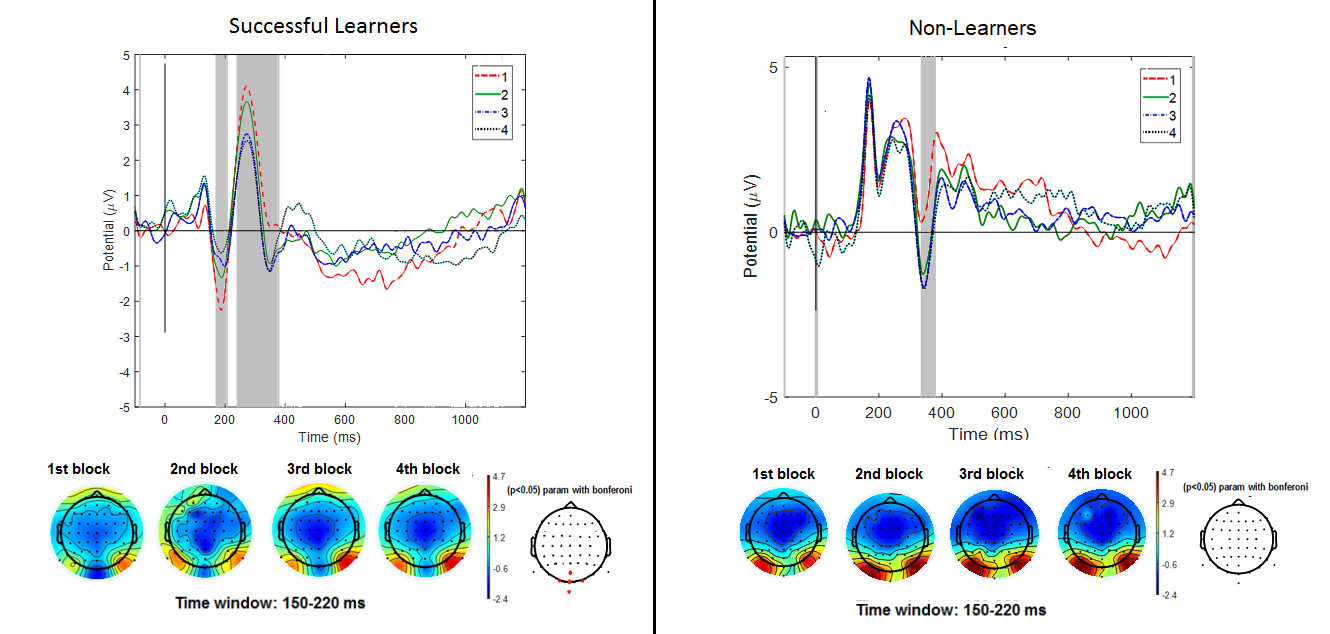

Supplement: S3 Fig — (TIF) [file pone.0226000.s004.tif]

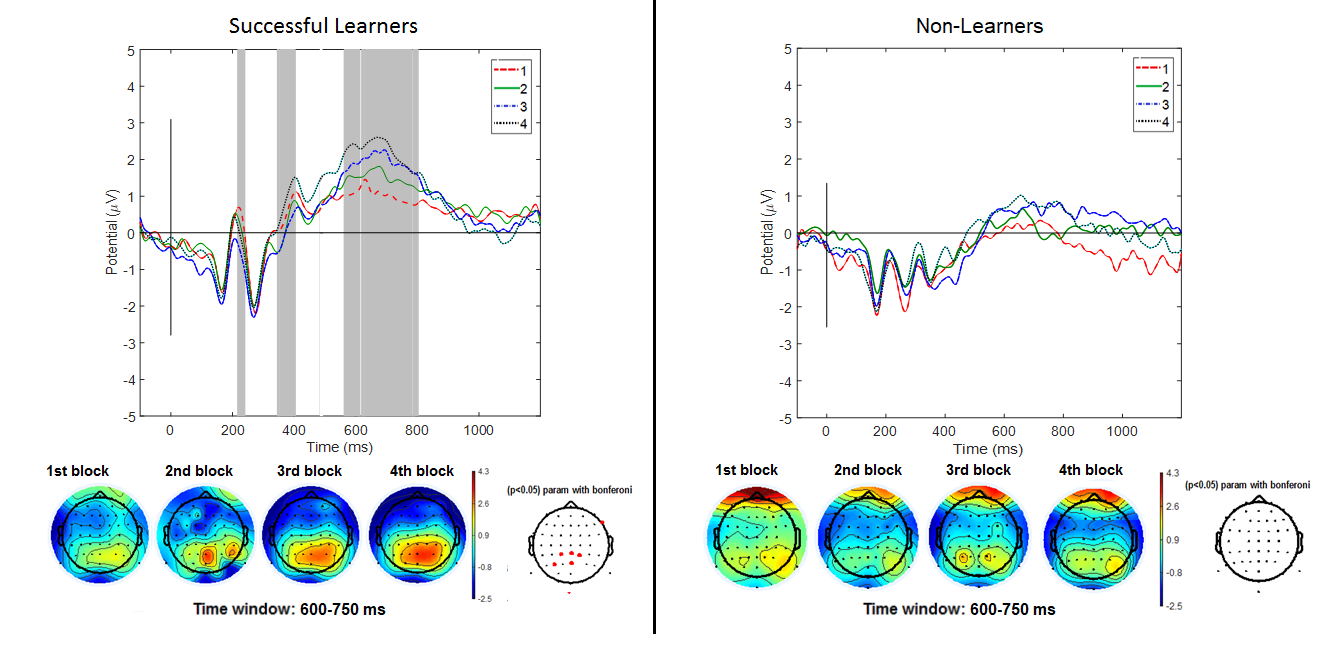

Supplement: S4 Fig — (TIF) [file pone.0226000.s005.tif]
